# Book Review: Language Learning Motivation: An Ethical Agenda for Research

**DOI:** 10.3389/fpsyg.2021.666289

**Published:** 2021-03-26

**Authors:** Xu Yang, Honggang Liu

**Affiliations:** School of Foreign Languages, Northeast Normal University, Changchun, China

**Keywords:** language learning motivation, ethical agenda, research ethics, social perspective, social values

To begin, ethical agenda in applied linguistics is “addressing real-world problems relating to language and communication in society” (Ushioda, 2020, p.16). It has been treated as the equivalent of ethical issues in doing research in applied linguistics, which at first restricted our understanding of the essence of this crucial term. Though it began to attract the attention in applied linguistics (e.g., Ortega, 2005; De Costa, 2015), much has still been neglected. In her book, Prof. Ushioda critically views the ethical agenda based on her ample research experiences in L2 motivation, and she developed this book to bring us fresh understandings of how to apply the ethical agenda to research practices.

Followed by an introductory chapter (Chapter 1), Chapters 2 to 6 critically examine a series of ethical issues in L2 motivation research, including the purposes of motivation research; how motivation theories conceptualize “language learners”; the social contexts focused on and neglected in the field and the language ideologies implicated in these contexts; the ethical issues in the implications for pedagogy and practice drawn from the research; and the ethical issues in the actual research practices. Based on the discussion in previous chapters, Ushioda articulates an ethical agenda for language motivation research in Chapter 7. In the concluding chapter (Chapter 8), the book explores some possible research frameworks and approaches for socially responsive motivation research. Ushioda advocates adopting teacher-researcher collaborative frameworks and teacher-led research inquiry, such as action research and exploratory practice.

According to Ortega (2005), the values of research are core to the ethical dimensions in social science research and should be evaluated by its “social utility” (p.430). A social perspective offers a potential avenue for conducting more socially meaningful applied linguistics research (Pérez-Milans, 2016). L2 motivation research celebrated its 60th birthday in 2019. However, ethical agenda, as a concept, is still left to be further explored in this field with regard to its implications for research perspective and practice.

The ethical agenda articulated makes it possible to investigate individuals' language learning motivation without isolating it from social ecology. Under the impact of the field of motivational psychology, the L2 motivation field tends to position the social and political contexts merely as background settings in research, which gives rise to a conceptual separation between the individual and the context. The tendency to separate the individual from external social contexts implicates the way researchers position the individual learners in research, namely viewing individual learners as abstractions consisting of a bundle of variables. In Chapter 3, Ushioda illustrates the “person-in-context relational view” of language learning motivation, emphasizing that individual learners are people in the real world and their language learning motivations are constantly changing in specific social realities. Ushioda argues for motivation research to link individuals' local realities with their language learning experiences, which can avoid the ethical risk of applying generalized and one-dimension implications for practice in highly diversified language learning contexts.

Furthermore, the ethical agenda has three main implications for motivation research practice. First, it emphasizes that research should not treat participants as “mice”; instead, it should bring about a win-win situation for the academic community and the community being investigated. However, L2 motivation research to date mainly serves the interests and needs of the academic community in pursuing theory development and knowledge advancement. According to Ushioda, although there are some implications for language classroom practice, these implications raise ethical questions about power in the classroom. Thus, in Chapter 5, Ushioda evaluates the ethics of motivating vs. controlling or manipulating students' behaviors in the language classroom and advocates that both L2 motivation researchers and practitioners have the responsibility to reflect on the motivational pedagogies they promote or implement. The second implication is that ethical issues need to be considered throughout the research project, especially during fieldwork research. For example, in a research project investigating language learning and teaching activities in the classroom, Ushioda found some comments on a questionnaire page, such as “Nobody wants to be friends with me,” and she then faced the ethical dilemma of choosing to tell the teacher about the child's difficulties out of moral concern or to protect the child's confidentiality, sticking to the traditional ethical protocol for research practice. The difficulty of dealing with such ethical dilemmas has not been mentioned in previous studies. Ushioda gives us a reminder in this respect, highlighting that ethical issues exist throughout research projects. It is also necessary to reflect on the ethical issues after the research process, as researchers have an ethical responsibility to reflect on the potential impacts of the research on participants and their motivations, especially the potentially negative impacts. The third, and final, implication of this research practice is that the ethical agenda points out that L2 motivation research should focus on more diversified language learning contexts. Research areas in applied linguistics have been characterized by a dominant focus on English learning contexts and university settings. Ushioda assumes that these relatively restricted contexts may implicitly promote an instrumentalist language ideology and elitist bias in the research. Even though she believes that it is difficult to identify which social contexts or communities the research needs to focus on most, we think paying attention to socially disadvantaged or marginalized groups and to language learning contexts lacking social support will make the ethical agenda more likely to bring about positive change to society. The ethical agenda in this book should be seriously treated by other arenas in applied linguistics for the social feature of this big field.

## Author Contributions

XY: drafts and revision. HL: revision and supervision. All authors: contributed to the article and approved the submitted version.

## Conflict of Interest

The authors declare that the research was conducted in the absence of any commercial or financial relationships that could be construed as a potential conflict of interest.
